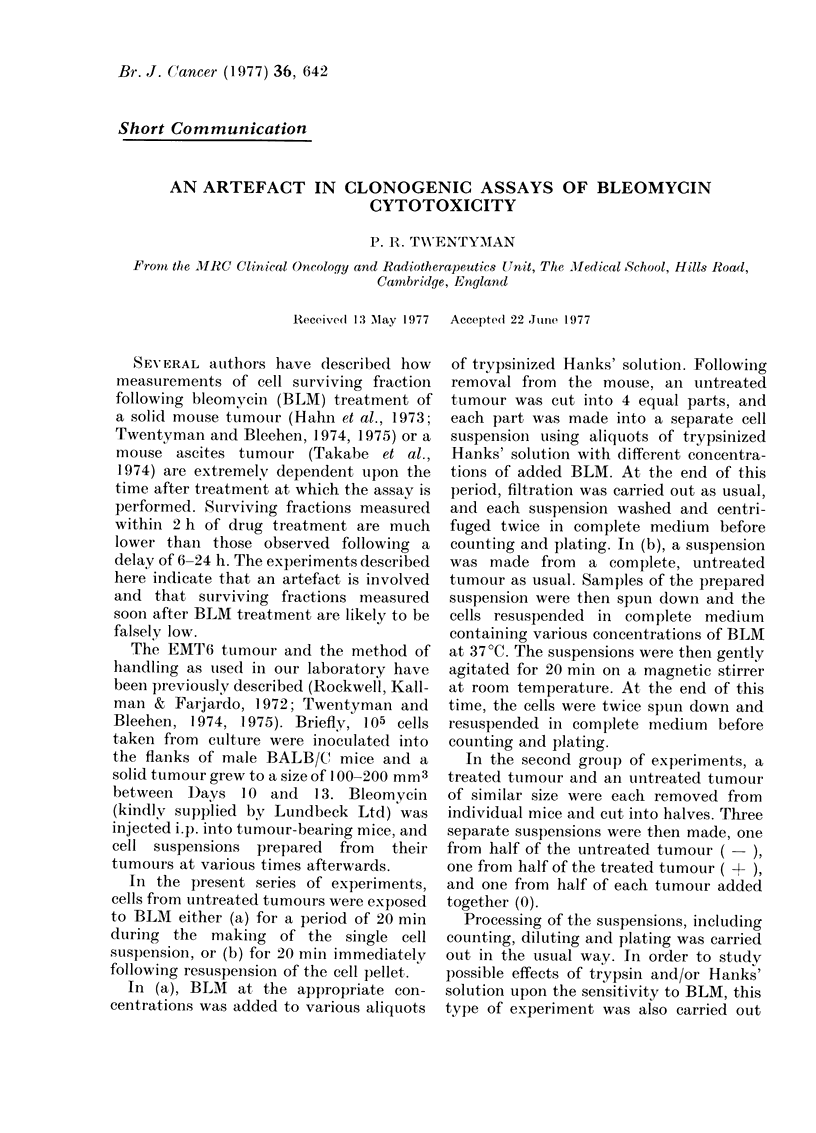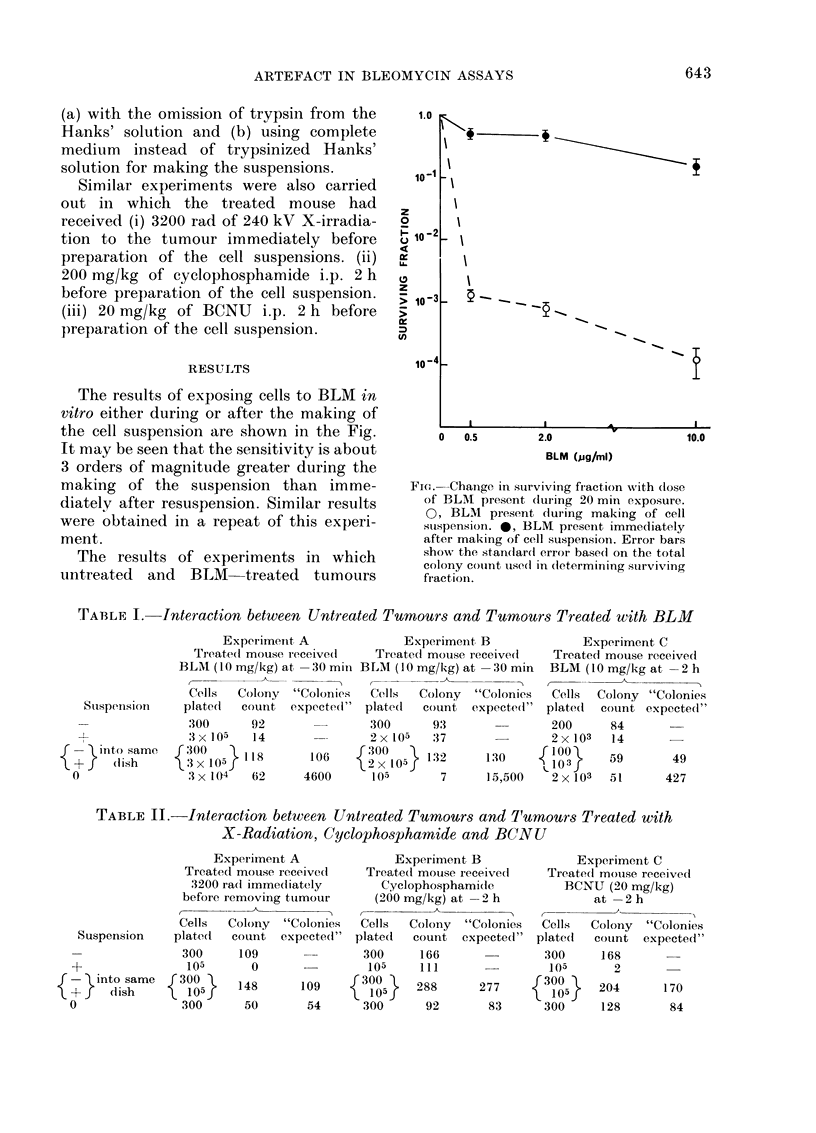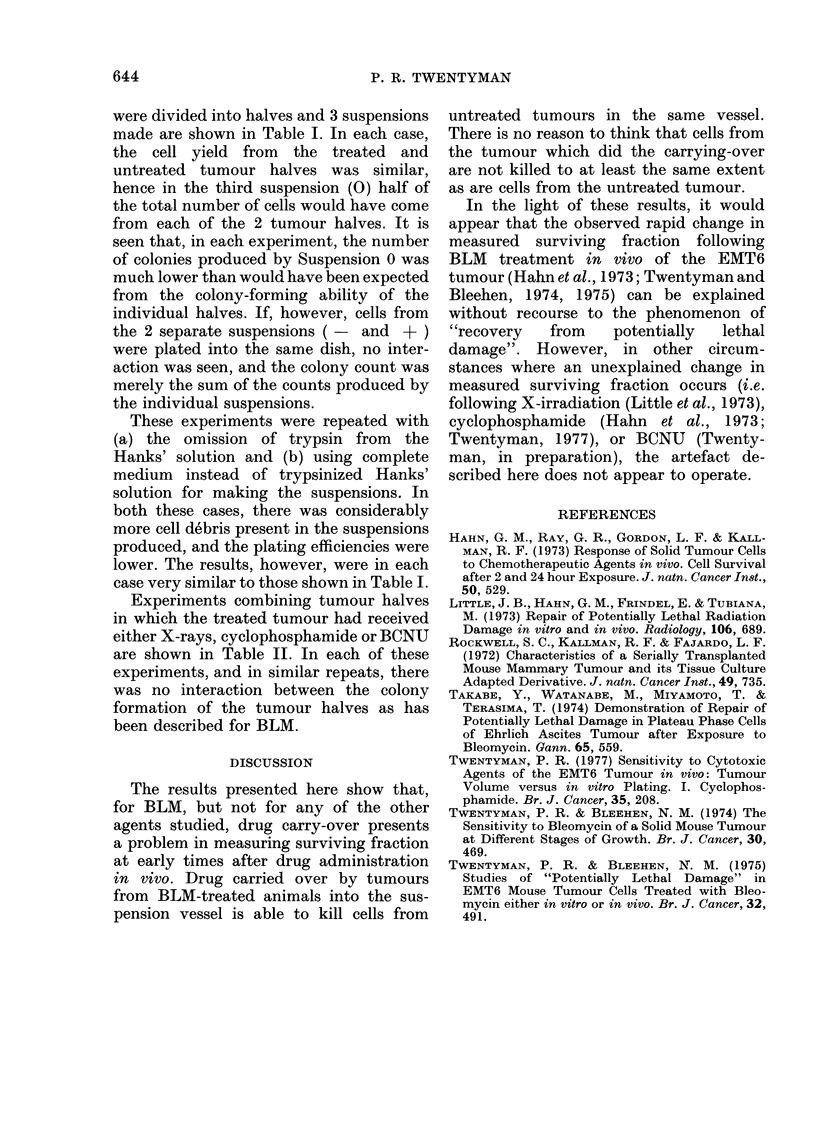# An artefact in clonogenic assays of bleomycin cytotoxicity.

**DOI:** 10.1038/bjc.1977.243

**Published:** 1977-11

**Authors:** P. R. Twentyman


Br. J. Cancer (1977) 36, 642

*Short Communication*

### AN ARTEFACT IN CLONOGENIC ASSAYS OF BLEOMYCIN CYTOTOXICITY

P. R. TWENTYMAN

*From the MRC Clinical Oncology and Radiotherapeutics Unit, The Medical School, Hills Road, Cambridge, England*

Received 13 May 1977 Accepted 22 June 1977

SEVERAL authors have described how measurements of cell surviving fraction following bleomycin (BLM) treatment of a solid mouse tumour (Hahn *et al.*, 1973; Twentyman and Bleehen, 1974, 1975) or a mouse ascites tumour (Takabe *et al.*, 1974) are extremely dependent upon the time after treatment at which the assay is performed. Surviving fractions measured within 2 h of drug treatment are much lower than those observed following a delay of 6–24 h. The experiments described here indicate that an artefact is involved and that surviving fractions measured soon after BLM treatment are likely to be falsely low.

The EMT6 tumour and the method of handling as used in our laboratory have been previously described (Rockwell, Kallman & Farjardo, 1972; Twentyman and Bleehen, 1974, 1975). Briefly, $10^5$ cells taken from culture were inoculated into the flanks of male BALB/C mice and a solid tumour grew to a size of 100–200 $mm^3$ between Days 10 and 13. Bleomycin (kindly supplied by Lundbeck Ltd) was injected i.p. into tumour-bearing mice, and cell suspensions prepared from their tumours at various times afterwards.

In the present series of experiments, cells from untreated tumours were exposed to BLM either (a) for a period of 20 min during the making of the single cell suspension, or (b) for 20 min immediately following resuspension of the cell pellet.

In (a), BLM at the appropriate concentrations was added to various aliquots of trypsinized Hanks' solution. Following removal from the mouse, an untreated tumour was cut into 4 equal parts, and each part was made into a separate cell suspension using aliquots of trypsinized Hanks' solution with different concentrations of added BLM. At the end of this period, filtration was carried out as usual, and each suspension washed and centrifuged twice in complete medium before counting and plating. In (b), a suspension was made from a complete, untreated tumour as usual. Samples of the prepared suspension were then spun down and the cells resuspended in complete medium containing various concentrations of BLM at 37 °C. The suspensions were then gently agitated for 20 min on a magnetic stirrer at room temperature. At the end of this time, the cells were twice spun down and resuspended in complete medium before counting and plating.

In the second group of experiments, a treated tumour and an untreated tumour of similar size were each removed from individual mice and cut into halves. Three separate suspensions were then made, one from half of the untreated tumour ( — ), one from half of the treated tumour ( + ), and one from half of each tumour added together (0).

Processing of the suspensions, including counting, diluting and plating was carried out in the usual way. In order to study possible effects of trypsin and/or Hanks' solution upon the sensitivity to BLM, this type of experiment was also carried out

(a) with the omission of trypsin from the Hanks' solution and (b) using complete medium instead of trypsinized Hanks' solution for making the suspensions.

Similar experiments were also carried out in which the treated mouse had received (i) 3200 rad of 240 kV X-irradiation to the tumour immediately before preparation of the cell suspensions. (ii) 200 mg/kg of cyclophosphamide i.p. 2 h before preparation of the cell suspension. (iii) 20 mg/kg of BCNU i.p. 2 h before preparation of the cell suspension.

### RESULTS

The results of exposing cells to BLM *in vitro* either during or after the making of the cell suspension are shown in the Fig. It may be seen that the sensitivity is about 3 orders of magnitude greater during the making of the suspension than immediately after resuspension. Similar results were obtained in a repeat of this experiment.

The results of experiments in which untreated and BLM—treated tumours

FIG.—Change in surviving fraction with dose of BLM present during 20 min exposure. ○, BLM present during making of cell suspension. ●, BLM present immediately after making of cell suspension. Error bars show the standard error based on the total colony count used in determining surviving fraction.

TABLE I.—*Interaction between Untreated Tumours and Tumours Treated with BLM*

| | Experiment A Treated mouse received BLM (10 mg/kg) at −30 min | | | Experiment B Treated mouse received BLM (10 mg/kg) at −30 min | | | Experiment C Treated mouse received BLM (10 mg/kg at −2 h | | |
|---|---|---|---|---|---|---|---|---|---|
| Suspension | Cells plated | Colony count | "Colonies expected" | Cells plated | Colony count | "Colonies expected" | Cells plated | Colony count | "Colonies expected" |
| − | 300 | 92 | — | 300 | 93 | — | 200 | 84 | — |
| + | $3\times10^5$ | 14 | — | $2\times10^5$ | 37 | — | $2\times10^3$ | 14 | — |
| {−, +} into same dish | {300, $3\times10^5$} | 118 | 106 | {300, $2\times10^5$} | 132 | 130 | {100, $10^3$} | 59 | 49 |
| 0 | $3\times10^4$ | 62 | 4600 | $10^5$ | 7 | 15,500 | $2\times10^3$ | 51 | 427 |

TABLE II.—*Interaction between Untreated Tumours and Tumours Treated with X-Radiation, Cyclophosphamide and BCNU*

| | Experiment A Treated mouse received 3200 rad immediately before removing tumour | | | Experiment B Treated mouse received Cyclophosphamide (200 mg/kg) at −2 h | | | Experiment C Treated mouse received BCNU (20 mg/kg) at −2 h | | |
|---|---|---|---|---|---|---|---|---|---|
| Suspension | Cells plated | Colony count | "Colonies expected" | Cells plated | Colony count | "Colonies expected" | Cells plated | Colony count | "Colonies expected" |
| − | 300 | 109 | — | 300 | 166 | — | 300 | 168 | — |
| + | $10^5$ | 0 | — | $10^5$ | 111 | — | $10^5$ | 2 | — |
| {−, +} into same dish | {300, $10^5$} | 148 | 109 | {300, $10^5$} | 288 | 277 | {300, $10^5$} | 204 | 170 |
| 0 | 300 | 50 | 54 | 300 | 92 | 83 | 300 | 128 | 84 |

were divided into halves and 3 suspensions made are shown in Table I. In each case, the cell yield from the treated and untreated tumour halves was similar, hence in the third suspension (O) half of the total number of cells would have come from each of the 2 tumour halves. It is seen that, in each experiment, the number of colonies produced by Suspension 0 was much lower than would have been expected from the colony-forming ability of the individual halves. If, however, cells from the 2 separate suspensions ( — and + ) were plated into the same dish, no interaction was seen, and the colony count was merely the sum of the counts produced by the individual suspensions.

These experiments were repeated with (a) the omission of trypsin from the Hanks' solution and (b) using complete medium instead of trypsinized Hanks' solution for making the suspensions. In both these cases, there was considerably more cell débris present in the suspensions produced, and the plating efficiencies were lower. The results, however, were in each case very similar to those shown in Table I.

Experiments combining tumour halves in which the treated tumour had received either X-rays, cyclophosphamide or BCNU are shown in Table II. In each of these experiments, and in similar repeats, there was no interaction between the colony formation of the tumour halves as has been described for BLM.

### DISCUSSION

The results presented here show that, for BLM, but not for any of the other agents studied, drug carry-over presents a problem in measuring surviving fraction at early times after drug administration *in vivo*. Drug carried over by tumours from BLM-treated animals into the suspension vessel is able to kill cells from untreated tumours in the same vessel. There is no reason to think that cells from the tumour which did the carrying-over are not killed to at least the same extent as are cells from the untreated tumour.

In the light of these results, it would appear that the observed rapid change in measured surviving fraction following BLM treatment *in vivo* of the EMT6 tumour (Hahn *et al.*, 1973; Twentyman and Bleehen, 1974, 1975) can be explained without recourse to the phenomenon of "recovery from potentially lethal damage". However, in other circumstances where an unexplained change in measured surviving fraction occurs (*i.e.* following X-irradiation (Little *et al.*, 1973), cyclophosphamide (Hahn *et al.*, 1973; Twentyman, 1977), or BCNU (Twentyman, in preparation), the artefact described here does not appear to operate.

### REFERENCES

HAHN, G. M., RAY, G. R., GORDON, L. F. & KALLMAN, R. F. (1973) Response of Solid Tumour Cells to Chemotherapeutic Agents *in vivo*. Cell Survival after 2 and 24 hour Exposure. *J. natn. Cancer Inst.*, **50**, 529.

LITTLE, J. B., HAHN, G. M., FRINDEL, E. & TUBIANA, M. (1973) Repair of Potentially Lethal Radiation Damage *in vitro* and *in vivo*. *Radiology*, **106**, 689.

ROCKWELL, S. C., KALLMAN, R. F. & FAJARDO, L. F. (1972) Characteristics of a Serially Transplanted Mouse Mammary Tumour and its Tissue Culture Adapted Derivative. *J. natn. Cancer Inst.*, **49**, 735.

TAKABE, Y., WATANABE, M., MIYAMOTO, T. & TERASIMA, T. (1974) Demonstration of Repair of Potentially Lethal Damage in Plateau Phase Cells of Ehrlich Ascites Tumour after Exposure to Bleomycin. *Gann*. **65**, 559.

TWENTYMAN, P. R. (1977) Sensitivity to Cytotoxic Agents of the EMT6 Tumour *in vivo*: Tumour Volume versus *in vitro* Plating. I. Cyclophosphamide. *Br. J. Cancer*, **35**, 208.

TWENTYMAN, P. R. & BLEEHEN, N. M. (1974) The Sensitivity to Bleomycin of a Solid Mouse Tumour at Different Stages of Growth. *Br. J. Cancer*, **30**, 469.

TWENTYMAN, P. R. & BLEEHEN, N. M. (1975) Studies of "Potentially Lethal Damage" in EMT6 Mouse Tumour Cells Treated with Bleomycin either *in vitro* or *in vivo*. *Br. J. Cancer*, **32**, 491.